# Breastfeeding and HIV: experiences from a decade of prevention of postnatal HIV transmission in sub-Saharan Africa

**DOI:** 10.1186/1746-4358-5-10

**Published:** 2010-10-26

**Authors:** Karen Marie I Moland, Marina M de Paoli, Daniel W Sellen, Penny van Esterik, Sebalda C Leshabari, Astrid Blystad

**Affiliations:** 1 Centre for International Health, University of Bergen, Norway; 2 Faculty of Health and Social Sciences, Bergen University College, Norway; 3 Fafo Institute for Applied International Studies, Oslo, Norway; 4 Department of Anthropology, University of Toronto, Canada; 5 Department of Anthropology, York University, Canada; 6 Muhimbili University of Health and Allied Sciences (MUHAS), Tanzania; 7 Department of Public Health and Primary Health Care, University of Bergen, Norway

## Abstract

Infant feeding by HIV-infected mothers has been a major global public health dilemma and a highly controversial matter. The controversy is reflected in the different sets of WHO infant feeding guidelines that have been issued over the last two decades. This thematic series, 'Infant feeding and HIV: lessons learnt and ways ahead' highlights the multiple challenges that HIV-infected women, infant feeding counsellors and health systems have encountered trying to translate and implement the shifting infant feeding recommendations in different local contexts in sub-Saharan Africa. As a background for the papers making up the series, this editorial reviews the changes in the guidelines in view of the roll out of prevention of mother to child transmission (PMTCT) programmes in sub-Saharan Africa between 2001 and 2010.

## Introduction

The papers in this thematic series highlight the multiple challenges to infant feeding that have surfaced within the framework of postnatal prevention of mother to child transmission of HIV (PMTCT). The focus is in particular on the implementation of the global HIV and infant feeding guidelines at the local level in various settings in sub-Saharan Africa. The symposium which preceded this publication dwelt on lessons learnt through the past decade of experiences with the implementation of the PMTCT programme. The symposium took place between 2 and 4 September 2008, in Rosendal, western Norway and was an interdisciplinary endeavour with students and scholars from eastern and southern Africa, Canada and Norway. The event offered an opportunity for dissemination of results from Masters, PhD and post-doctoral studies on the PMTCT challenge. The seven research papers included in this issue report on the experiences of women and their partners, of PMTCT counsellors and of policy makers in selected countries in sub-Saharan Africa between 2001 and 2009. During this period the WHO 2001 HIV and infant feeding guidelines [1] were launched and implemented. These guidelines which provide concrete recommendations on 'safer infant feeding options' were to become very influential in the following decade.

## Discussion

### Mother to child transmission of HIV

Mother to child transmission (MTCT) of HIV is a field of health and health care that dramatically demonstrates the inequality between the global north and the global south. The best available official data indicate that "more than 90% of children living with HIV acquired the virus during pregnancy, birth or breastfeeding -- forms of HIV transmission that can be prevented" [[2] p. 37]. The epidemiology and the burden of disease vary greatly across regions [3], and nearly 90% of the almost half a million children who yearly become infected with HIV through their mothers live in sub-Saharan Africa [2]. The region has the highest indicators of food insecurity, highest prevalence of adult and paediatric HIV infection, the highest incidence of AIDS and AIDS-related morbidity and death, and the highest proportion of HIV and AIDS affected households [2]. While interventions have reduced the MTCT rate to less than 2% in high income settings [4,5], it remains unacceptably high in low income settings varying between 20-45% [6]. If no precautions are taken, about one third of the children born to HIV-infected mothers will contract the virus, and it is estimated that transmission through breastfeeding accounts for one third to one half of these infections [6].

With the current knowledge and technology most cases of postnatal MTCT are preventable through antiretroviral (ARV) drugs and modifications in infant feeding practices. During the last decade an increasing number of HIV-infected women have gained access to antiretroviral treatment or prophylaxis effectively reducing transmission during pregnancy and birth [7], but the transmission of HIV through breastfeeding has remained a challenge in contexts where breastfeeding is normative and vital to infant survival. Breastfeeding transmission has in fact come to contribute to an increasing part of the total MTCT in the region [3].

### Standard PMTCT programmes

The initial standard PMTCT programme package which was implemented in most sub-Saharan settings between 2001 and 2009 was exclusively preventive. The sole aim of all interventions including medication of the mother was to prevent the baby from acquiring HIV. The services included voluntary and later routine counselling and testing (VCT/RCT), different regimens of ARV prophylaxis to mother and child around the time of birth, and infant feeding counselling based on the 2001 WHO guidelines [1]. The major infant feeding options for HIV-infected mothers suggested in local PMTCT programmes in sub-Saharan Africa included exclusive breastfeeding with early and rapid cessation (at six months) and exclusive replacement feeding with infant formula or modified cow's milk.

### The evolving evidence base on infant feeding methods

The issue of infant feeding has been particularly challenging in a PMTCT context because the options available, breastfeeding or not breastfeeding, both involve risks to child health and survival. How to weigh the benefits of breastfeeding against the risk of HIV infection is an issue that has been vigorously debated. The dilemma becomes particularly acute since the greatest proportion the global burden of HIV and AIDS lies in sub-Saharan Africa where the major causes of infant death are malnutrition and infectious diseases [8]. Breastfeeding involves a considerable risk of HIV transmission but at the same time not breastfeeding represents a considerable risk to infant survival in low-income countries [9]. A WHO meta-analysis in 2000 showed that infants who were not breastfed and received infant feeding formula or other replacement food had a 6-fold increased risk of dying during the first two months of life, a 4-fold increased risk between two to three months, and a 2.5-fold increased risk between four and five months compared with those who were breastfed [10]. The authors of the study concluded that "it will be difficult, if not impossible, to provide safe breast milk substitutes to children from underprivileged populations"[[10] p. 455]. This resulted in renewed attention to the complexity of dangers related to the use of infant feeding formula in low-income settings, and to the importance of breastfeeding promotion for child survival internationally [11].

Within PMTCT programmes, research on how to make breastfeeding safer identified the increased risks of mixed feeding [12]. The customary extended breastfeeding pattern with early supplementation of fluids and solids, so-called mixed feeding, was documented to strongly increase the risk of HIV transmission to the baby [6,12,13]. Presumably the mechanism behind this is that additional liquids and fluids may compromise intestinal integrity and result in small lesions in the immature gut where the virus can pass to infect the infant [12]. Several studies have shown that exclusive breastfeeding, i.e. giving only breast milk and no other liquids or solids, reduces the transmission rate to less than one fourth compared to mixed feeding [12-14]. Still more importantly, subsequent studies demonstrated that exclusive breastfeeding increased the survival chances of HIV exposed infants. In a randomized trial Thior and colleagues found that even if breastfeeding with ARV prophylaxis (zidovudine) was not as effective as formula feeding in preventing postnatal HIV transmission, breastfeeding was associated with a lower infant mortality rate at seven months [15]. These findings were supported by the large intervention cohort study conducted by Coovadia and colleagues in South Africa [13] which documented a higher HIV-free survival among the exclusively breastfed than among the replacement-fed infants. The study found that infants who were exclusively breastfed did not run a greater postnatal risk of HIV transmission than infants who were formula fed, and that mortality at three months was almost doubled in the group who received infant formula compared with the exclusively breastfed infants.

### The history of the WHO infant feeding guidelines

The WHO HIV and infant feeding guidelines have been shifting with developments in knowledge and technology. The WHO in collaboration with UNAIDS and UNICEF have to our knowledge produced 16 documents, possibly more, on infant feeding guidelines for HIV-infected women over the last quarter century [1,10,16-29]. These documents have been developed in response to an evolving evidence-base on the risk of HIV transmission through breastfeeding, mixed feeding and the potential risk of malnutrition and increased vulnerability to gastrointestinal and respiratory tract infections associated with replacement feeding. HIV was discovered in human milk and linked to paediatric infection in 1985 [30-32]. In the initial phase of the epidemic the probability of HIV transmission through breastfeeding was still uncertain and thought to be relatively small. Breastfeeding by biological mother was thus recommended to be the feeding method of choice irrespective of HIV status in economically disadvantaged settings. In 1992, a WHO consultation concluded that "Where infectious diseases and malnutrition are the main cause of infant deaths and the infant mortality rate is high, breastfeeding should be the usual advice given to pregnant women including those who are HIV-infected" [[33] p. 178].

In 1997-98 the WHO published new infant feeding guidelines which advised that all mothers should be counselled about possible feeding options and thus be allowed to make their own decision about infant feeding. This was interpreted as a major policy shift towards the promotion of replacement feeding. Based on this shift the distribution of free infant formula to HIV positive women enrolled in PMTCT programmes was initiated in some countries [8].

With the rapidly emerging evidence of the challenges of replacement feeding in low income contexts the WHO, in 2001 [1], accommodated the possibility that exclusive breastfeeding could be safer than mixed feeding, and that replacement feeding represented a risk to child health and survival. The so called AFASS criteria [1,21] were introduced in an attempt to bring the local context of infant feeding and the circumstances of the individual mother systematically into the decision making process. The recommendation now read: "when replacement feeding is acceptable, feasible, affordable, sustainable, and safe (AFASS), avoidance of all breastfeeding by HIV-infected mothers is recommended. Otherwise, exclusive breastfeeding for the first few months of life is recommended" [[1] p. 12], but should be discontinued as soon as feasible. The feeding options recommended for HIV-infected mothers in the 2001 guidelines consisted of the following options: replacement feeding with commercial infant formula or modified animal milk, exclusive breastfeeding with early and rapid cessation at six months, expressed heat-treated breast milk or wet-nursing by an HIV-negative mother, the latter two receiving less attention due to their perceived local inapplicability.

According to this guideline the decision about whether or not to breastfeed should be made by every mother based on full information of the options available. The guiding principle was that HIV-infected mothers should be supported in making an informed decision about how to best feed their infants [21]. This positioned the counsellor as an informed expert possessing the knowledge and authority to enable individual mothers to make an 'informed choice' of an infant feeding method.

Although the 2001 guidelines brought in the AFASS criteria which promoted an assessment of each woman's situation and choice, they still strongly communicated that replacement feeding was the first choice of infant feeding method for HIV positive women, and that exclusive breastfeeding should be discontinued as soon as replacement feeding was AFASS. Major challenges have been that both exclusive breastfeeding and replacement feeding have been found to be extremely difficult to adhere to for the large majority of the HIV-infected mothers [34-36], and the AFASS criteria have proven to be very difficult to operationalize at the local programmatic level [37].

In 2006, after strong evidence of the risks of childhood infections and malnutrition associated with replacement feeding, and with the path-breaking documentation of a higher HIV free survival rate among exclusively breastfed than among replacement fed infants [13], the WHO infant feeding guidelines were updated [38]. The AFASS criteria were still emphasised, but the wording slightly changed placing exclusive breastfeeding first: "Exclusive breastfeeding is recommended for HIV-infected mothers for the first six months of life unless replacement feeding is acceptable, feasible, affordable, sustainable and safe (AFASS) for them and their infants before that time; When replacement feeding is acceptable, feasible, affordable, sustainable and safe, avoidance of all breastfeeding by HIV-infected mothers is recommended" [[38] p. 9]. The update launched a major policy shift on breastfeeding cessation, and moved away from rapid cessation at six months. It stated that if replacement feeding is not AFASS at six months, the mother should continue to breastfeed while giving complementary foods until a nutritionally adequate and safe diet without breast milk could be provided.

In 2009, WHO launched the so-called Rapid Advice [28] building on the evidence of HIV free infant survival and on new research showing that ARV interventions for HIV-infected mothers or HIV-exposed infants can significantly reduce the risk of transmission of HIV through breastfeeding. Access to ARV treatment proved to have major implications for the safety of breastfeeding in HIV-infected women. The Rapid Advice was quickly followed by the 2010 HIV and infant feeding guidelines [29]. The key principles related to postnatal HIV transmission currently are the following: balancing HIV prevention with protection from other causes of child mortality; informing mothers known to be HIV-infected about infant feeding alternatives (individual rights should not be forfeited in the course of public health approaches); providing services to support mothers to appropriately feed their infants; avoiding harm to infant feeding practices in the general population [29]. The concrete recommendations in these latest guidelines are the following [29]:

• Mothers known to be HIV-infected should exclusively breastfeed their infants for the first six months of life, introducing appropriate complementary food thereafter, and continue breastfeeding for the first 12 months of life;

• Mothers who decide to stop breastfeeding should stop gradually within one month; stopping breastfeeding abruptly is not advisable;

• Mothers known to be HIV-infected should only give commercial infant formula milk as a replacement feed to their HIV uninfected infants or infants who are of unknown status, when specific conditions are met (referred to as AFASS);

• Mothers known to be HIV-infected should be provided with lifelong antiretroviral therapy or antiretroviral prophylaxis interventions.

The 2001 and the 2010 guidelines each produced in response to the existing evidence available, stand in sharp contrast to one another both in terms of the recommended first choice of feeding method (2001 replacement feeding; 2010 exclusive breastfeeding); breastfeeding cessation (2001 rapid cessation; 2010 complementary feeding) and in terms of the principle of informed choice (2001 individual informed choice; 2010 national or sub-national level choice). It is however predominantly the 2001 version promoting replacement feeding as the first choice and exclusive breastfeeding only if replacement feeding is not AFASS that has informed training and infant feeding counselling in PMTCT programmes across sub-Saharan Africa during the past decade. The HIV epidemic coupled with the assumed benefits of infant feeding formula for all HIV-infected mothers have in complex ways changed public ideas about infant feeding, and has introduced an important threat to well established breastfeeding practices.

The pace, frequency, and characteristics of the changes have been influenced by continuing weaknesses in the scientific understanding of the mechanisms and risks of infection, but also by failure in early political leadership to demand timely funding to conduct research to properly address these weaknesses [39]. The suite of changes, inadequacies, inconsistencies, failures and unintended implications of the changing evidence base and policy framework have made it extremely challenging to produce timely and consistent policy for implementing effective national PMTCT programmes. The concomitant shifts in postnatal PMTCT guidelines have been extremely challenging for health systems to implement, and have thus significantly hampered their effectiveness [39-42].

The history of the shifting WHO guidelines on HIV and infant feeding in the period 1998-2010 can be seen as a history of retreat from recommendations based on narrow medical research and on ideologies of individual choice, to recommendations recognising the importance of local social and cultural context as well as local knowledge on breastfeeding and survival. Most significantly the shifts in infant feeding recommendations in the 2010 guidelines promoting exclusive breastfeeding for six months as the method of choice, and complementary feeding after six months are thus more in line with the public health recommendations to the general population. They are also more in line with customary infant feeding practices in sub-Saharan Africa. The move away from replacement feeding as first choice brings breastfeeding back as the prime way of feeding an infant and implies a major gain in public health. Moreover it is expected that the new formulation will also protect HIV-infected women from suspicion and stigma attached to non-normative infant feeding practices. In the long run the most important outcome will be to protect the general public against spill-over effects from the replacement feeding promotion that indirectly has taken place through PMTCT infant feeding counselling [8].

### The papers

The papers included here look back on the past decade of PMTCT implementation and discuss the lessons learnt. They reveal different dimensions of the PMTCT guidelines and how they enter into action across national and local settings. They present glimpses of the intricate challenges encountered by the targets of global policy guidelines - the HIV-infected mothers, the PMTCT counsellors, local policy makers and peer supporters. The papers add to and challenge the strictly defined biomedical evidence, and bring to the forefront experience-based and context bound knowledge.

The problems involved in translating the 2001 WHO HIV and infant feeding guidelines to national policy and programming are dealt with in Chinkonde *et al*.'s paper from Malawi [43]. In a context where the WHO infant feeding recommendation were found to be non-applicable to the vast majority of Malawian HIV-infected mothers, various stakeholders engaged in a search for feasible solutions. While HIV-related actors argued in terms of HIV-free survival, others argued primarily in terms of infant nutrition and infant survival. The process stranded, with no national recommendation document for PMTCT programmes and counsellors, creating grounds for poor quality and haphazard counselling of the mothers [43]. The lack of coherent national recommendations has had repercussions for Malawi's response to later shifts in the global HIV and infant feeding guidelines. The continuing challenge for policy makers described in the paper is to keep up-to-date with substantial and frequent changes in recommendations created at the global level.

The confusion in relating to the guidelines is more concretely revealed in the powerful accounts of HIV-infected mothers who have to handle their own HIV positive status while at the same time struggling with the possibility of transmitting the virus to their infant through breastfeeding. Koricho *et al*.'s paper describes the confusion and fear that Ethiopian mothers face trying to adhere to the advice given by the nurse-counsellors [44]. The intense experience among many HIV-positive mothers of having 'poisonous milk' led to desperate attempts to secure infant formula among mothers who were too poor to buy enough. Many of the women would thus eventually resort to breastfeeding when they ran out of cash leading to experiences of guilt and deep regret. The fear of transmitting HIV to the infants also dominated the messages of the PMTCT counsellors who commonly ended up recommending that breastfeeding should be avoided if at all possible [44].

The lack of local applicability of the PMTCT guidelines is also revealed concretely in Engebretsen *et al*.'s paper from Uganda where both exclusive breastfeeding and exclusive formula feeding were deemed inappropriate and unsatisfactory by the study participants [45]. The paper describes the deep-seated cultural significance of breast milk as the only culturally appropriate way to feed an infant. This message was communicated in talks where men were active participants in the debates about the feeding of their children. The paper, however, reveals the lack of appropriate arenas for men to engage in such debates, the PMTCT field being dominated by a 'mother and child' approach (inherent in the name) [45]. Such lack of partner involvement in the infant health sphere has emerged as perilous in an HIV context where modification of infant feeding regimes is highly dependent upon male support.

The acknowledgement of the importance of partner involvement in PMTCT programmes has led to a strong call for partner disclosure. A woman's disclosure of positive HIV status to her partner at the vulnerable time of pregnancy or after birth has however been experienced as highly problematic. Njunga and Blystad's paper describes the implications of partner disclosure within a matrilineal and matrilocal kinship structure in Malawi [46]. Men in these settings move to women's villages at marriage. With the revelation of an HIV positive diagnosis by his wife, the husband will as the one 'moving into his wife's family' be looked upon as the one who brought the infection to his wife's clan and kin. The experience of blame for infecting the family of his in-laws will commonly be so difficult to live with that husbands eventually leave their wives and children to manage on their own. The PMTCT programme has locally been named 'the divorce programme', vividly expressing the local dynamics at work [46].

At the core of the 2001 guidelines lies the concept of early and rapid cessation of exclusive breastfeeding at six months. But extended breastfeeding and mixed feeding is normative in large parts of the world. Levy *et al*'s study from Malawi demonstrates the many obstacles of emotional, practical and economic kind that HIV-infected women experience in their attempts to wean their children abruptly and at a very early age [47]. Mothers struggle with the guilt of not breastfeeding and tend to see the survival of the baby as their sole responsibility. Levy *et al*. describes the demands of the PMTCT programme as a 'downloading of responsibility' onto HIV-positive mothers who are unable to secure the baby a safe transition to other foods without support [47].

The modifications of customary infant feeding practices required in order to adhere to the 2001 WHO guidelines have been very complex and challenging. There has been a growing recognition that without support for the women who must relate to difficult infant feeding regimes there is little chance that they will succeed. A number of different initiatives of support have been launched in the past decade and include projects like mother to mother support groups, home based care, peer education and peer counselling. Nankunda *et al*.'s study from Uganda describes how highly the women in the study valued the support for exclusive breastfeeding from the peer educators and how this became decisive for their ability to adhere to exclusive breastfeeding [48]. The peer educators being mothers themselves, their careful approaches, and the fact that they returned many times to visit the mothers in their homes were raised as particularly central dimensions for the success in guiding women through the six months period of exclusively breastfeeding their children [48].

However, peer PMTCT support programmes as well as other support initiatives operate in a context fraught with tension and fear. The peer counsellors are left with the challenge of convincing HIV-infected mothers that either exclusive breastfeeding or exclusive replacement feeding is a safe and feasible way of feeding a baby and of reducing the risk of HIV transmission. This is not an easy task. Nkonki and Daniels's paper from South Africa demonstrates how the counsellors struggled to 'sell their service' to a sceptical group of potential recipients through a continuous negotiation of their own credibility [49]. Nkonki and Daniels describe the stress of long days of travelling where mothers commonly remained doubtful of counsellors' messages [49].

## Conclusions

The papers together highlight some of the key challenges that emerged in the wake of WHO's 2001 HIV and infant feeding guidelines. They reveal the complexity of dilemmas and the adverse effects that can be generated by global policy guidelines that are very distant from local lives.

The WHO infant feeding guidelines have changed considerably since the studies in this issue were carried out. In light of new evidence, operational experiences and increased availability of ARV both for prophylaxis and treatment, one may ask how relevant the results in the present papers are to current and future PMTCT programme implementation. Recent guidelines have a broader approach than the 2001 version. The protection of maternal prenatal and postnatal health, viral load and CD4 status have been included in addition to HIV-free survival of the child [28]. The infant feeding recommendations are also dramatically altered. Despite such radical changes, it remains a legacy of the decade that the 2001 guidelines became extremely influential as they coincided with the large scale roll-out of the PMTCT programme, and were fundamental in the training of a generation of postnatal PMTCT counsellors. It is most probable that the ambiguous policy on breastfeeding launched in these guidelines will have long lasting repercussions for public health efforts on infant feeding in sub-Saharan Africa for years to come. It may take years for national programmes and health services to overcome the confusions created in the wake of the WHO's 2001 infant feeding recommendations. It may take even longer to return breastfeeding to its social position as the "the only way to feed an infant" [34], as a condition for child survival and as a fundamental commitment of motherhood [36].

## Competing interests

The authors declare that they have no competing interests.

## Authors' contributions

KMM, AB and MDP wrote the first draft of the paper. DS contributed to subsequent drafts. PVE was a key person during the workshop and wrote a summing up paper that was drawn upon in the introduction. SCL commented on later drafts. All authors approved the final paper.
